# The efficacy and safety of peripheral intravenous parenteral nutrition vs 10% glucose in preterm infants born 30 to 33 weeks’ gestation: a randomised controlled trial

**DOI:** 10.1186/s12887-020-02280-w

**Published:** 2020-08-17

**Authors:** Hiroki Suganuma, Dennis Bonney, Chad C. Andersen, Andrew J. McPhee, Thomas R. Sullivan, Robert A. Gibson, Carmel T. Collins

**Affiliations:** 1 SAHMRI Women and Kids, South Australian Health and Medical Research Institute Adelaide, South Australia, Australia; 2 Discipline of Paediatrics, Adelaide Medical School, The University of Adelaide, Adelaide, SA Australia; 3 Neonatal Medicine, Women’s and Children’s Hospital, Adelaide, SA Australia; 4 School of Public Health, The University of Adelaide, Adelaide, SA Australia; 5 School of Agriculture Food and Wine, The University of Adelaide, Adelaide, SA Australia

**Keywords:** Preterm infant, Parenteral nutrition, Intravenous lipids

## Abstract

**Background:**

Preterm infants born 30 to 33 weeks’ gestation often require early support with intravenous fluids because of respiratory distress, hypoglycemia or feed intolerance. When full feeds are anticipated to be reached within the first week, risks associated with intravenous delivery mode and type must be carefully considered. Recommendations are for parenteral nutrition to be infused via central venous lines (because of the high osmolarity), however, given the risks associated with central lines, clinicians may opt for 10% glucose via peripheral venous catheter when the need is short-term. We therefore compare a low osmolarity peripheral intravenous parenteral nutrition (P-PN) solution with peripheral intravenous 10% glucose on growth rate in preterm infants born 30 to 33 weeks’ gestation.

**Methods:**

In this parallel group, single centre, superiority, non-blinded, randomised controlled trial, 92 (P-PN 42, control 50) infants born 30^+ 0^ to 33^+ 6^ weeks’ gestation, were randomised within 24 h of age, to receive either P-PN (8% glucose, 30 g/L amino acids, 500 IU/L heparin and SMOFlipid®) or a control of peripheral intravenous 10% glucose. Both groups received enteral feeds according to hospital protocol. The primary outcome was rate of weight gain from birth to 21 days of age.

**Results:**

The rate of weight gain was significantly increased in P-PN infants compared with control (P-PN, *n* = 42, 18.7, SD 6.6 g/d vs control, *n* = 50, 14.8, SD 6.0 g/d; adjusted mean difference 3.9 g/d, 95% CI 1.3 to 6.6; *P* = 0.004), with the effect maintained to discharge home. Days to regain birthweight were significantly reduced and length gain significantly increased in P-PN infants. One infant in the P-PN group had a stage 3 extravasation which rapidly resolved. Blood urea nitrogen and triglyceride levels were significantly higher in the P-PN group in the first week of life, but there were no instances of abnormally high levels. There were no significant differences in any other clinical or biochemical outcomes.

**Conclusion:**

P-PN improves the rate of weight gain to discharge home in preterm infants born 30 to 33 weeks gestation compared with peripheral intravenous 10% glucose.

**Trial registration:**

Australian New Zealand Clinical Trials Registry ACTRN12616000925448. Registered 12 July 2016.

## Background

Infants born 30 to 33 weeks’ gestation constitute approximately 2–3% of the infant population and a large proportion of neonatal admissions. This population of preterm infants often requires transient respiratory support, are at risk of hypoglycemia and feed intolerance such that intravenous fluids are often provided during the early stages of their care. This early neonatal period corresponds to a critical window during which under-nutrition may have long lasting effects on growth and development. Lower intelligence quotient and more attention and behavioral problems at school age are evident not only in very preterm infants [1] but also in moderately preterm infants [2–4] when compared with infants born at term. We, and others, have shown that in-hospital growth is related to later developmental outcome and that improvements in growth rate are associated with better mental development [5, 6].

Although recommendations for early parenteral amino acids and lipid support for very preterm infants (< 32 weeks’ gestation) are clear [7, 8], the nutritional requirements of the moderately preterm infant (32 to 33 weeks’ gestation) are less well established [9]. While practices vary, in many centres moderately preterm infants receive 10% glucose using a peripheral intravenous cannula in the first week of life as enteral feeds are established [10, 11]. Parenteral nutrition solutions are typically given via central venous catheters due to their high osmolarity and risks with extravasation if delivered peripherally [12–15]. However, central venous lines are not without risk and consequently infants requiring them are cared for in intensive care settings [12, 13]. Most moderately preterm newborns [10, 11], and in our experience even many less mature infants (30 to 31 weeks’ gestation), do not get central venous catheters as they are considered physiologically stable enough to be able to tolerate full enteral nutrition by 5–7 days of age and thus can be cared for in Special Care Units [10, 11]. Although recent recommendations state that peripheral venous parenteral nutrition can be given for short periods, the level of evidence for this is low and the risks associated with extravasation high [13, 14]. In addition recommendations state that peripheral venous delivery should only be used when the osmolarity of the infusate is < 850–900 mOsm/L [13, 15].

Consequently, these infants receive less protein and lipid nutrition in the first week of life in comparison to both in-utero accretion and their more immature ex-utero counterparts. Clinicians therefore are balancing the risks associated with the use of central venous lines to deliver parenteral nutrition with the risks associated with short term poorer nutrition as full enteral feeds are established. We therefore aimed to determine the efficacy and safety of providing peripherally administered, low osmolarity, intravenous parenteral nutrition to preterm infants born 30 to 33 weeks’ gestation.

## Methods

### Study design

The study was a single centre (Women’s and Children’s Hospital, North Adelaide, South Australia), parallel group, superiority, randomised controlled trial conducted between September 2016 and June 2018. The trial protocol was approved by the Human Research Ethics Committee (HREC/15/WCHN/134) of the Women’s and Children’s Hospital and the study was registered with the Australian New Zealand Clinical Trials Registry (ACTRN12616000925448). The study adheres to CONSORT guidelines for reporting of randomised controlled trials [16].

### Participants

Infants born 30^+ 0^ to 33^+ 6^ weeks’ gestation at the Women’s and Children’s Hospital who required intravenous fluids and were less than 24 h of age and whose parents were able to provide informed written consent, were eligible to participate. Multiple births were eligible and were randomised individually. Infants receiving fluids administered centrally or presenting with major congenital or chromosomal abnormalities were ineligible. Infants were required to be enrolled and randomised before 24 h of age.

### Randomisation and blinding

Infants were randomised to one of two groups: the peripheral parenteral nutrition (P-PN) group or control (peripheral 10% glucose) with a 1:1 allocation according to a computer-generated randomisation schedule developed by an independent statistician. Originally the schedule was to be stratified by sex and gestational age 30^+ 0^ to 31^+ 6^ and 32^+ 0^ to 33^+ 6^ weeks’ using permuted blocks of random sizes. Unfortunately, during development, the sequence of randomisations within each stratum was unintentionally re-sorted according to a randomly generated uninformative study identifier; this was not discovered until the study was complete. This essentially nullified the effects of blocking and meant the final randomisation procedure most closely approximated simple randomisation.

Parents of eligible infants were approached by a clinician (medical practitioner or neonatal nurse practitioner) and followed-up for consent by a research nurse who was not involved in clinical care. Upon consent, infants were randomised by a research nurse or clinician using REDCap (Research Electronic Data Capture) - a secure web-based software platform hosted at the South Australian Health and Medical Research Institution [17, 18]. Data analysts were blinded to group allocation. It was not possible to blind families, clinicians and the researchers who conducted data collection.

### Interventions

The intervention P-PN solution was prepared by Baxter Healthcare and contained amino acids (as Primene®) 30 g/L, glucose 80 g/L (8%) and heparin 500 IU/L. The P-PN solution was provided in 400 mL bags and had an estimated osmolality of 678 mOsm/L. The intervention was given to a maximum of 100 mL/kg/d, so the infant received a maximum intravenous protein intake of 3 g/kg/d. If additional parenteral fluid was required to maintain the targeted total fluid volume or for physiological homeostasis, 10% glucose was given as a separate line via the same peripheral intravenous site. The lipid solution was a 17% lipid emulsion with added vitamins for administration (SMOFlipid® 20% 15 mL, Vitalipid N Infant® 4 mL and Soluvit N® 1 mL per 20 mL, Fresenius Kabi) with the estimated osmolality of 340 mOsm/L. The lipid emulsion (with vitamins) was administered using a separate line via the same peripheral intravenous site at 2 g/kg/d and was included in the total amount of intravenous daily fluids. For the period prior to randomisation and commencement of the intervention solutions, infants received intravenous 10% glucose via peripheral venous catheter.

The control group received peripheral intravenous 10% glucose (osmolarity 556 mOsm/L) administered as per the Women’s and Children’s Hospital neonatal fluid management guidelines. Electrolytes were added, if clinically indicated, using a commercially available premixed solution (Glucose 100 g/L, Potassium chloride 1.5 g/L, Sodium chloride 2.25 g/L; osmolarity 672 mOsm/L).

The fluid management approach was the same for both groups and followed the Hospital guidelines with total fluid volume commenced at 60 mL/kg/d, increasing by 10–15 mL/kg/d to 150–170 ml/kg/d. Enteral feeds (typically expressed breast milk, EBM, or less commonly preterm formula when EBM not available) are commenced when clinically stable. EBM is typically fortified when the enteral intake is > 80 mL/kg/d. The IV infusions ceased in both the intervention and control groups when an enteral intake of 120 mL/kg/d was reached and maintained for 3 days. Lipid emulsion was administered at 2 g/kg/d and ceased at an enteral intake of 100 mL/kg/d. Fluid balance records were audited daily for compliance with the trial protocol. Peripheral venous cannulae were routinely changed every 72 h.

### Outcome assessments

The primary outcome was weight gain (g/d) from birth to 21 days ±2 days. Body weight was measured by clinical staff at approximately the same time daily using electronically balanced scales. Secondary efficacy outcomes included: weight (g/d), length and head circumference gain (mm/d) from birth to discharge home; weight, length and head circumference at 21 days of age ± 2 days and on discharge home. Length was measured weekly and on day of discharge home by clinical staff using a recumbent length board measured to the nearest 0.1 cm. Head circumference was measured around the largest occipitofrontal circumference, using a non-stretching tape, weekly and on day of discharge home by clinical staff. Secondary safety outcomes included extravasation stage 3 or 4 [19], the number of infants requiring central venous catheter insertion, duration of peripheral venous cannula, feeding tolerance (the number of days on which one or more feeds were stopped) and the number of days taken to reach enteral intake ≥120 mL/kg/d and maintained for 3 days. Clinical outcomes included confirmed sepsis, days of any respiratory support and length of hospital stay (collected according to the Australian and New Zealand Neonatal Network data definitions) [20].

Protein, lipid and energy intake over the first 21 days were assessed. Parenteral and enteral intake data were collected prospectively from fluid balance charts. The macronutrient composition of the intravenous solutions and formula were based on manufacturer information, and human milk on published values [21]. Energy intake was calculated using the Atwater factors of 4, 4 and 9 kcal per gram of protein, carbohydrate and fat, respectively. Blood samples were taken to assess protein and lipid safety on study day 1, 2, 4, 7, 14 and 21. Blood urea nitrogen (BUN), albumin, triglycerides, pH, base excess and blood glucose levels were measured at hospital laboratories. All data were entered into REDCap [17, 18].

### Sample size and statistical analysis

Assuming a standard deviation in weight gain of 5 g per day in this population [22], 45 infants per group (total of 90 infants) were required to detect a difference in weight gain of 3 g per day between groups with 80% power (*P* < 0.05). Consultation with the neonatal medical team agreed that this was a clinically important difference on which clinical practice would change.

All analyses were carried out on an intention to treat basis according to a pre-specified statistical analysis plan. Weight gain from birth to 21 days was compared between groups using linear regression, with adjustment made for sex and gestational age at birth (30^+ 0^ to 31^+ 6^ and 32^+ 0^ to 33^+ 6^ weeks) and generalised estimating equations used to account for clustering due to multiple births. Secondary efficacy and safety outcomes were compared between groups using linear, logistic, and negative binomial regression models as appropriate, again using generalised estimating equations to account for clustering due to multiple births. Secondary biochemical measures obtained from the blood samples and weight z-scores were compared between groups over time using linear mixed models, with fixed effects terms for group, time and the interaction between group and time included in each model. For the primary outcome only, a per-protocol analysis including only those infants whose clinical care adhered to the study protocol (i.e. received P-PN and control solutions via peripheral line) was also undertaken. We calculated z-scores for weight using Australian standards [23]. All analyses were performed using R 3.5.1 (R Core Team, 2019) [24].

## Results

### Trial population

**Fig. 1:**
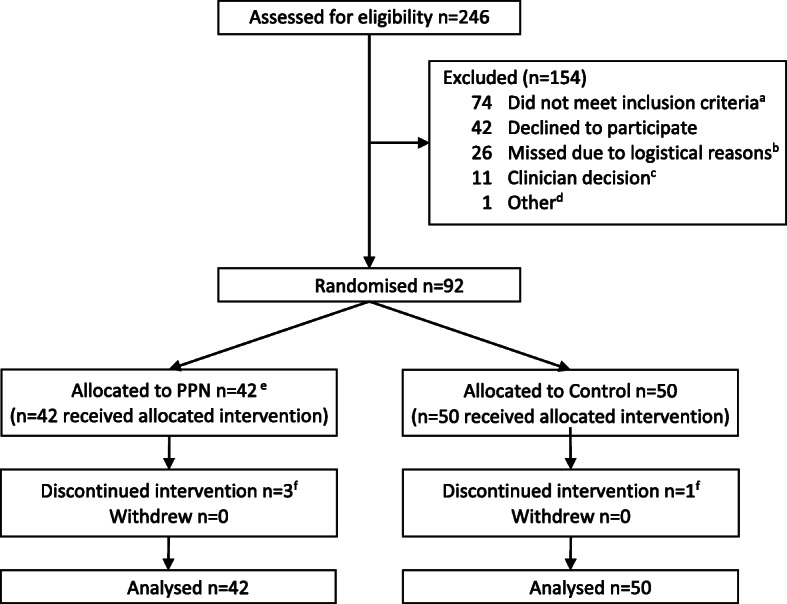
Participant flow through the study. ^a^ Did not meet gestational age criteria *n* = 2, Out born *n* = 13, Central line placed, or anticipated to be placed, within 24 h *n* = 35, Congenital or chromosomal abnormality *n* = 7, > 24 h of age *n* = 1, Language difficulty *n* = 7, Parent < 18 years of age *n* = 2, Did not require IV *n* = 1, IV for < 24 h *n* = 1, Imminent transfer to stepdown hospital *n* = 3, Died *n* = 1, Insufficient study product available *n* = 1. ^b^ Staff not available *n* = 26. ^c^ Anticipated to reach full enteral feeding within 3 days *n* = 10, given glucagon *n* = 1. ^d^ No decision made by the parents within 24 h *n* = 1. ^e^ One out born infant randomised in error and included in all analyses. ^f^ Discontinued the study fluids due to clinical condition and need for central line insertion – commenced standard PN and SMOFlipid®

Ninety-two infants were enrolled in the study with 42 infants randomised to the P-PN group and 50 infants to the control (Fig. 1). In total, four infants (P-PN 3, control 1) required central venous line insertion due to their clinical condition and commenced ‘standard preterm PN’ [25] (Baxter Healthcare Pty Ltd) and SMOFlipid®. All 92 infants were included in intention-to-treat analyses with 88 infants included in the per-protocol analysis. Baseline demographic and clinical characteristics were similar between the groups, although there were more singleton infants randomised to the control group than the P-PN group (Table 1 and Supplementary Table 1, Additional File).

**Table 1:** Baseline demographic and clinical characteristics

**Variable**	**P-PN (*n*** **= 42)**	**Control (*n*** **= 50)**
**Infant characteristics**		
Female sex	17 (40)	17 (34)
Gestational age, median (IQR), wks	32 (31–32)	32 (31–33)
30^+ 0^–31^+ 6^ weeks’ gestation	15 (36)	19 (38)
32^+ 0^–33^+ 6^ weeks’ gestation	27 (64)	31 (62)
Singleton	18 (43)	32 (64)
Twins	18 (43)	15 (30)
Triplets	6 (14)	3 (6)
Apgar at 5 min, median (IQR) (*n* = 41/50)	9.0 (8.0–9.0)	8.5 (8.0–9.0)
Birth weight, mean (SD), g	1717 (289)	1749 (329)
Birth weight z-score, mean (SD)	−0.2 (0.7)	−0.1 (0.7)
Birth length, mean (SD), cm	42 (2)	42 (2)
Birth head circumference, mean (SD), cm	30 (2)	30 (2)
**Maternal characteristics**		
Maternal age, mean (SD), yr	32 (5)	31 (5)
Vaginal birth	14 (33)	15 (30)
Caesarean section	28 (67)	35 (70)
Antenatal steroids - any	24 (57)	35 (70)

Data are presented as n (%) unless otherwise indicated

### Nutritional management

All infants received peripheral intravenous 10% glucose until randomisation. Infants randomised to the P-PN group commenced the intervention solutions at a median of 18 h of age (IQR 11–26 h). The number of days requiring intravenous therapy were similar between groups (P-PN 5.9, SD 1.9 d, control 5.7, SD 1.3 d; adjusted ratio of means 1.0, 95% CI 0.9 to 1.1, *P* = 0.5). Infants randomised to P-PN had significantly higher parenteral protein, lipid and energy intake in week 1 (Supplementary Table 2, Additional File). There were no significant differences in enteral protein, lipid or energy intake between groups over the three-week study period (Supplementary Table 2, Additional File).

### Primary outcome

Infants randomised to P-PN had a significantly greater rate of weight gain from birth to day 21 compared with infants randomised to control (P-PN 18.7, SD 6.6 g/d vs control 14.8, SD 6.0 g/d; adjusted mean difference 3.9 g/d, 95% CI 1.3 to 6.6; *P* = 0.004) (Table 2). The effect was similar (P-PN 19.3, SD 6.3 g/d vs control 14.8, SD 6.1 g/d; adjusted mean difference 4.4 g/d, 95% CI 1.9 to 7.03; *P* = 0.0008) when analysed per protocol, i.e. excluding the 4 infants who required a central line and received ‘standard preterm PN’ [14] and SMOFlipid® (Table 2).

**Table 2:** Growth outcomes

**Outcome**	**P-PN (*n*** **= 42)**	**Control (*n*** **= 50)**	**Adjusted mean difference (95% CI)**	**Adjusted** ***P*** **value**^**a**^
Weight gain from birth to day 21, g/d	18.7 (6.6)	14.8 (6.0)	3.9 (1.3 to 6.6)	0.004
*Per protocol* weight gain from birth to day 21, g/d^b^	19.3 (6.3)	14.8 (6.1)	4.4 (1.9 to 7.0)	0.0008
Birthweight regained, d, (*n* = 41/49)	9.8 (2.9)	12.3 (2.8)	0.8 (0.7 to 0.9)^c^	< 0.0001
Weight gain from birth to discharge home, g/d, (*n* = 42/49)	24.1 (5.3)	19.4 (8.4)	4.9 (2.0 to 7.8)	0.001
Length gain from birth to discharge home, mm/d, (*n* = 39/48)	1.2 (0.5)	1.0 (0.7)	0.3 (0.0 to 0.5)	0.02
Head circumference gain from birth to discharge home, mm/d, (*n* = 39/47)	0.9 (0.4)	0.9 (0.4)	0.1 (−0.1 to 0.2)	0.4
Weight at day 21, g^d^	2087 (332)	2042 (340)	76 (24 to 129)	0.004
Length at day 21 ± 2 days, cm, (*n* = 28/34)^d^	44.5 (2.8)	43.4 (2.1)	1.3 (0.5 to 2.1)	0.001
Head circumference at day 21 ± 2 days, cm, (*n* = 28/34)^d^	31.0 (2.0)	31.0 (1.4)	0.2 (−0.3 to 0.6)	0.5
Weight on discharge home, g, (*n* = 42/49)^d^	2561 (328)	2463 (359)	129 (4 to 254)	0.05
Length on discharge home, cm, (*n* = 39/48)^d^	45.8 (2.3)	45.3 (2.2)	0.7 (0.2 to 1.5)	0.1
Head circumference on discharge home, cm, (*n* = 39/47)^d^	32.8 (1.2)	32.8 (1.7)	0.1 (0.5 to 0.7)	0.7

Data are presented as mean (SD)

^a^ Adjusted for sex and gestational age 30^+ 0^ to 31^+ 6^ and 32^+ 0^ to 33^+ 6^ weeks

^b^ Per protocol analysis included infants whose clinical care adhered to the study protocol, i.e. received P-PN and Control via peripheral line: P-PN *n* = 39, Control *n* = 49

^c^ Adjusted ratio of means

^d^ Additionally adjusted for corresponding anthropometric measure at birth

### Secondary outcomes

#### Growth

**Fig. 2:**
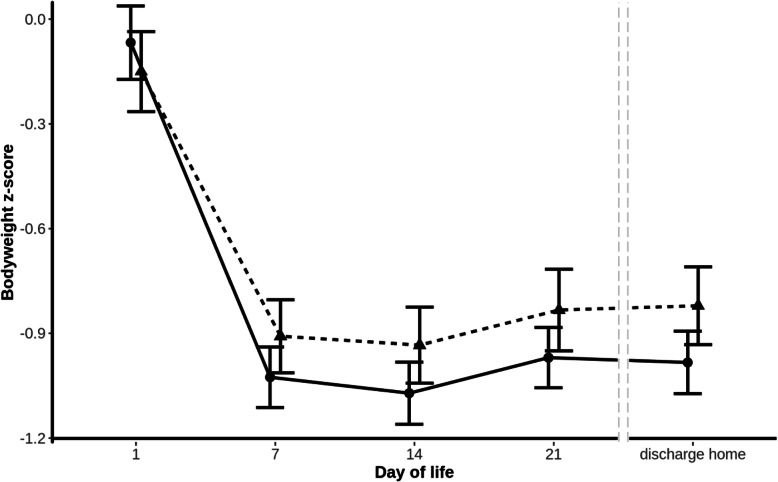
Body weight z-scores. Broken line and triangle shape represents the P-PN intervention group, and solid line and closed circle represents the Control group. The figure represents means and standard error of the mean. The overall interaction effect *P* value = 0.7 and the overall adjusted mean difference is 0.2 (0.04, 0.3), *P* = 0.008 (adjusted for sex, gestational age and baseline weight z-score). P-PN/Control: Day 1 *n* = 42/50, Day 7 *n* = 42/50, Day 14 *n* = 40/48, Day 21 *n* = 42/50, discharge 38/45

Infants randomised to P-PN regained birthweight significantly faster than infants randomised to control (adjusted ratio of means 0.8 days, 95% CI: 0.7 to 0.9; *P* < 0.0001) (Table 2). Weight and length at day 21 were significantly higher in the P-PN group compared with control (Table 2). By discharge home weight, but not length, remained significantly higher. There was no difference in head circumference at either time point (Table 2). On discharge home, the rate of weight gain from birth remained significantly greater in infants randomised to P-PN than those randomised to control (adjusted mean difference 4.9 g/d, 95% CI 2.0 to 7.8 g/d; *P* = 0.001). Length gain to discharge home was also significantly greater in P-PN infants compared with control (adjusted mean difference 0.3 mm/d, 95% CI 0.0 to 0.5 mm/d; *P* = 0.02), however there were no differences in rate of head circumference gain (Table 2). The P-PN group had an overall greater body weight z-score compared with control (adjusted mean difference 0.2, 95% CI 0.04 to 0.3; *P* = 0.008) (Fig. 2).

#### Clinical outcomes

There were no significant differences between groups in the proportion of infants treated for hypoglycemia or developing feeding intolerance, or in days taken to reach full enteral feeds (Table 3). There were no significant differences in any clinical outcomes including respiratory support requirements, incidence of sepsis and length of hospital stay (Table 3 and Supplementary Table 3, Additional File).

**Table 3:** Clinical outcomes

**Outcome**	**P-PN (*n*** **= 42)**	**Control (*n*** **= 50)**	**Adjusted effect**^**a**^ **(95% CI)**	**Adjusted** ***P*** **value**^**a**^
Days of feeding intolerance, mean, SD, d^b^	0.6 (1.2)	0.6 (1.0)	1.0 (0.5, 2.1)^c^	1.0
Days to full enteral feeds (120 mLs/kg/d), mean, SD, d	6.6 (2.4)	6.2 (1.7)	1.1 (0.9, 1.2)^c^	0.3
Hypoglycemia requiring treatment	4.0 (9.5)	8.0 (16.0)	0.5 (0.1, 2.0)^d^	0.4
IPPV via endotracheal tube	3.0 (7.1)	4.0 (8.0)	1.1 (0.2, 5.9)^d^	0.9
Nasal CPAP	26.0 (61.9)	37.0 (74.0)	0.6 (0.2, 1.5)^d^	0.3
Days of any respiratory support, mean, SD, d	4.2 (7.0)	5.3 (12.0)	0.8 (0.4, 1.5)^c^	0.5
Confirmed sepsis^e^	2 (4.8)	1 (2.0)	2.1 (0.2, 19.0)^d^	0.5
Breastmilk (any) on discharge home (*n* = 38/46)	33 (87)	41 (89)	0.8 (0.2, 3.3)^d^	0.8
Length of hospital stay, mean, SD, d	35.5 (10.5)	35.6 (13.8)	1.0 (0.9, 1.1)^c^	0.8

Data are presented as n (%) unless otherwise indicated

^a^ Adjusted for sex and gestational age 30^+ 0^ to 31^+ 6^ and 32^+ 0^ to 33^+ 6^ weeks

^b^ Number of days on which one or more feeds were stopped

^c^ Adjusted ratio of means

^d^ Adjusted odds ratio

^e^ P-PN group rhinovirus *n* = 1, central line sepsis *n* = 1; Control group rhinovirus *n* = 1

#### Biochemistry

There was a significant group by time interaction for both mean BUN and mean triglyceride levels (*P* < 0.0001) with BUN levels significantly increased to day 7 and triglyceride levels to day 14 in the P-PN group compared with control (Supplementary Table 5, Additional File). However, there were no instances of raised BUN levels (14.3 mmol/L [26]) in either group (Supplementary Table 6, Additional File). Hypertriglyceridemia (> 2.25 mmol/L [27]) occurred in four infants, one each in the P-PN and control group on days 2 and 7 (Supplementary Table 6, Additional File). There were no significant differences between the groups for mean levels of serum albumin, pH, base excess and blood sugar (Supplementary Table 5, Additional File) nor in proportion of infants with low serum albumin, hyperglycaemia or metabolic acidosis (Supplementary Table 6, Additional File).

#### Adverse events

Neither the overall length of time that peripheral venous cannulae were required, the frequency of infiltration nor the number of peripheral cannulae used differed between groups (Supplementary Table 4, Additional File). There was one stage 3 extravasation in the P-PN group which resolved quickly on removal of the cannula. There were no stage 3 or 4 extravasations in the control group.

## Discussion

In this single centre, randomised controlled trial, in preterm infants born 30 to 33 weeks’ gestation requiring intravenous fluids, peripheral intravenous parenteral nutrition (8% glucose, 30 g amino acids/L, heparin 500 IU/L) and SMOFlipid® resulted in a significantly greater rate of weight gain from birth to 21 days of age when compared with peripheral intravenous 10% glucose.

The increased rate of weight gain was maintained to discharge home. The time to regain birthweight in P-PN infants was significantly less than control infants with P-PN infants weighing significantly more at 21 days of age and on discharge home. Length gain from birth to 21 days and birth to discharge home were also significantly greater with the intervention, however, there was no effect on head circumference. We found no evidence of adverse effects relating to the P-PN intervention.

To the best of our knowledge this is the first randomised controlled trial of parenteral nutrition in this population. Previous observational reports show wide variation in clinical practice between countries and centres reflecting the lack of evidence in the nutritional management of this population [10, 11, 28–30]. Delivery of parenteral nutrition using a central line is common in some centers [29, 31] and has been reported to improve nutrient intake and postnatal growth [31]. Central lines allow infusion of high osmolarity parenteral nutrition however their use is not without clinical risk, for e.g., sepsis, haemorrhage, thrombosis, air leak syndromes [13, 32, 33]. Consequently, insertion and care of central lines requires particular expertise, necessitating admission to a neonatal intensive care setting. Caution regarding insertion of central lines in infants who are expected to reach full enteral feeds within ≈5–7 days may explain some of the variation in nutritional management practices. Results from a recent Australian and New Zealand survey [10] and a UK audit [11] in infants born 32–34 weeks’ gestation show that < 20% use parenteral nutrition in this population with resulting suboptimal nutrient intakes [11].

Our study is unique in that parenteral nutrition was administered via peripheral venous catheter, thus avoiding the use of a central line, and reducing need for intensive care, while maximising nutritional intake. Zecca et al. [34] studied a different approach to improving nutrition in their randomised controlled of a ‘proactive feeding regimen’ (enteral intake 100 mLs/kg/d day 1, increasing by day 3 to 200 mLs/kg/d) vs standard care (enteral intake 60 mLs/kg/d day 1, increasing by day 9 to 170 mL/kg/d). They showed a significant reduction in length of stay (mean, 9.8, SD 3.1 vs 11.9, SD 4.7 days; *P* = 0.03), need for intravenous fluids (2.8% vs 33.3%; *P* = 0.001) with no difference in feeding tolerance. However, their population was considerably more mature (32–36 weeks’ gestation) with the trial specifically designed for infants small for gestational age. Their approach may not translate to the less mature infant and such a study would need to be repeated in this population.

During the conduct of this study results from a large (*n* = 1440) RCT suggested that delaying the introduction of parenteral nutrition for 7 days is advantageous in critically ill children [35, 36]. Only 15% (*n* = 209) of participants in their study were neonates and all were term born, it is therefore unknown if this benefit would apply to infants with the degree of prematurity included in our study and who had mild transitional problems. Observational studies in the moderately preterm infant have shown an association between minimising postnatal weight losses and improved growth rate [37]. In the extremely preterm infant poor postnatal growth is not only associated with serious complications of prematurity such as bronchopulmonary dysplasia, necrotising enterocolitis and sepsis but also poorer neurodevelopment [5, 6, 38, 39]. Sufficiently powered randomised controlled trials will be required to detect differences in these less common clinical outcomes in the moderately preterm infant, and effects on longer term neurodevelopment.

Although a economic analysis was beyond the scope of this study, we acknowledge there is a marginal increase in costs associated with the use of intravenous parenteral nutrition compared with 10% glucose. However, the increase in growth we found will allow 30–33 week preterm infants to remain in hospitals providing Special Care only without need to transfer to a major perinatal center for intensive care with potential reduction in health care costs.

The peripheral parenteral nutrition solution used was specifically designed to have an osmolarity comparable to that of those fluids routinely used peripherally in our institution (< 700 mOsmol/L) thus minimising the risk of phlebitis and extravasation injuries associated with hyperosmolar solutions. The osmolarity of the available standardised formulations exceeded this range [25]. We achieved the reduction in osmolarity by reducing the glucose concentration to 8% to accommodate the addition of protein. The solution did not include additional electrolytes that may not be essential for the 30 to 33 week preterm newborn in the early days of life and which may increase the risk of extravasation injuries. The osmolarity of this infusate was calculated to be 678 mOsmol/L which sits within both the European [13] and North American [40] guidelines for peripheral administration and therefore could be used in both neonatal intensive and special care nurseries.

We found no adverse events associated with the intervention. Although there was one grade 3 extravasation in the intervention group this rapidly resolved on removal of the cannula without any further intervention. The parenteral nutrition and lipid intervention were well tolerated. While the BUN levels were higher in the first week of life in the P-PN infants than in the control there were no instances of abnormally high levels, nor was there any evidence of metabolic acidosis. Hypertriglycidaemia occurred in only one infant in each group, with the infant in the control group not having received intravenous lipids; and instances of both hypo- and hyperglycemia were similar between groups.

Our study was limited by not being able to blind the intervention. The study team considered many options for blinding such as having the intervention and control fluids masked within amber opaque syringes and infusion tubing. However, safety considerations were thought to be prohibitive for this strategy. Data analysts were blinded to group allocation and unblinding did not occur until all analyses according to the a priori statistical analysis plan were complete. The randomisation schedule error resulted in simple rather than blocked randomisation. While this led to a small imbalance in numbers between groups (P-PN 42, control 50), sex and gestational age strata were balanced between groups. A further limitation was that secondary outcomes were analysed without adjustment for multiple comparisons. Although treatment effects on secondary growth and biochemistry outcomes were clinically plausible and often highly statistically significant, the lack of multiplicity adjustment means these findings should be interpreted with additional caution.

## Conclusion

Providing peripherally administered parenteral nutrition of 8% glucose, 30 g/L amino acids and SMOFlipid® improves short-term weight and length gain in infants born 30–33 weeks’ gestation.

## Supplementary information

**Additional file 1: Supplementary Table 1.** Baseline characteristics. **Supplementary Table 2.** Parenteral and enteral intake over 21 day study period. **Supplementary Table 3.** Clinical outcomes. **Supplementary Table 4.** Peripheral intravenous cannula. **Supplementary Table 5.** Mean biochemical measures by day of life and overall. **Supplementary Table 6.** Biochemical measures outside normal clinical parameters.

## Data Availability

Deidentified individual participant data will be made available to researchers who provide a methodologically sound proposal for use in achieving the goals of the approved proposal. Proposals should be submitted to the corresponding author for review by the trial steering committee.

## Abbreviations

BUN: Blood urea nitrogen
IQR: Interquartile range
P-PN: Peripheral parenteral nutrition
SD: Standard deviation
