# Supplementary material for: The efficacy and safety of peripheral intravenous parenteral nutrition vs 10% glucose in preterm infants born 30 to 33 weeks’ gestation: a randomised controlled trial
Source: BMC Pediatr. 2020 Aug 17;20:384. doi: 10.1186/s12887-020-02280-w (PMC7429781; doi:10.1186/s12887-020-02280-w)
Supplement: Supplementary file 1 — Additional file 1: Supplementary Table 1. Baseline characteristics. Supplementary Table 2. Parenteral and enteral intake over 21 day study period. Supplementary Table 3. Clinical outcomes. Supplementary Table 4. Peripheral intravenous cannula. Supplementary Table 5. Mean biochemical measures by day of life and overall. Supplementary Table 6. Biochemical measures outside normal clinical parameters. [file 12887_2020_2280_MOESM1_ESM.pdf]

Additional File

This appendix has been provided by the authors to give readers additional information about their work.

Supplement to: The efficacy and safety of peripheral intravenous parenteral nutrition and lipids vs 10% glucose in preterm infants born 30 to 33 weeks' gestation: a randomised controlled trial.

Contents

Supplementary Table 1. Baseline characteristics 2
Supplementary Table 2. Parenteral and enteral intake over 21 day study period 3
Supplementary Table 3. Clinical outcomes 5
Supplementary Table 4. Peripheral intravenous cannulae 5
Supplementary Table 5. Mean biochemical measures by day of life and overall 6
Supplementary Table 6. Biochemical measures outside normal clinical parameters .. 8

Supplementary Table 1. Baseline characteristics

Variable	P-PN (n=42)	Control (n=50)
**Maternal**		
Caesarean section in labor	9 (21)	5 (10)
Caesarean section, no labor	19 (45)	30 (60)
Antenatal steroids – complete course	19 (45)	22 (44)
**Infant characteristics**		
Birth order		
Singleton	18 (43)	32 (64)
First of a multiple	9 (21)	11 (22)
Second of a multiple	14 (33)	5 (10)
Third of a multiple	1 (2)	2 (4)
Apgar score		
At 1 minute, median (IQR), (n=41/50)	7 (6 - 9)	7 (6 - 8)
Temperature		
On admission, mean, SD	36.4 (0.5)	36.4 (0.5)
Admission temperature ≤36.5°C	20 (48)	28 (45)
Admission temperature ≥36.5°C to ≤37.4°C	21 (50)	21 (42)
Admission temperature >37.4°C	1 (2)	1 (2)
Baseline biochemistry		
Blood urea nitrogen, mmol/L, mean (SD) (n=37/42)	4.6 (1.5)	4.5 (1.6)
Albumin, g/L, mean (SD) (n=41/41)	24.6 (3.2)	25.4 (3.0)
Triglyceride, mmol/L, median (IQR) (n=40/42)	0.6 (0.5-0.6)	0.6 (0.4-0.7)
pH, mean (SD) (n=42/47)	7.4 (0.0)	7.4 (0.1)
Base excess, mean (SD) (n=42/47)	-1.8 (1.7)	-2.3 (2.3)
Blood glucose level, mmol/L, mean (SD) (n=42/47)	4.8 (1.1)	4.4 (1.4)

Data are presented as n (%) unless otherwise indicated

Supplementary Table 2. Parenteral and enteral intake over 21 day study period

Outcome	P-PN (n=42)	Control (n=50)	Adjusted mean difference[a] (95% CI)	P[a]
**Week 1**				
Parenteral				
Protein (g/kg/d)	1.1 (0.4)	0.0 (0.1)	1.10 (1.0, 1.2)	<0.0001
Lipid (g/kg/d)	1.1 (0.3)	0.0 (0.2)	1.1 (1.0, 1.2)	<0.0001
Energy (kcal/kg/d)[b]	29.3 (9.5)	18.5 (6.6)	1.6 (1.4, 1.9)[c]	<0.0001
Enteral[d]				
Protein (g/kg/d) (n=41/50)	1.0 (0.5)	1.0 (0.5)	0.0 (-0.2, 0.2)	0.9
Lipid (g/kg/d) (n=41/50)	2.2 (0.9)	2.4 (0.8)	-0.1 (-0.5, 0.2)	0.5
Energy (kcal/kg/d)[b] (n=41/50)	41.2 (16.5)	42.8 (15.1)	0.9 (0. 7, 1.2)[c]	0.4
**Week 2**[e]				
Enteral [d]				
Protein (g/kg/d) (n=40/47)	3.3 (0.6)	3.4 (0.5)	-0.1 (-0.3, 0.2)	0.5
Lipid (g/kg/d) (n=40/47)	6.4 (0.9)	6.7 (0.6)	-0.3 (-0.7, 0.0)	0.09
Energy (kcal/kg/d)[b] (n=40/47)	124.5 (16.8)	129.5 (11.7)	1.0 (0.9, 1.0)[c]	0.08
**Week 3**[f]				
Enteral intake				
Protein (g/kg/d) (n=37/43)	3.6 (0.4)	3.5 (0.5)	0.1 (-0.1, 0.3)	0.3
Lipid (g/kg/d) (n=37/43)	6.7 (1.0)	6.7 (1.0)	0.0 (-0.3, 0.4)	0.9
Energy (kcal/kg/d) (n=37/43)	133.5 (15.2)	132.1 (10.4)	1.0 (1.0, 1.1)	0.7

Data are presented as mean (SD)

[a] Adjusted for sex and gestational age at birth

[b] Log-transformed due to heterogeneous residual variance

**Outcome**	**P-PN (n=42)**	**Control (n=50)**	**Adjusted mean difference[a] (95% CI)**	**P[a]**

[c] Effects are ratio of geometric means
[d] Intake data not collected because of transfer to another hospital or discharge home: PPN – Transferred week 1 n=1, week 2 n=1, week 3 n=1; Discharged home week 3 n=2; Control - Transferred week 2 n=2, week 3 n=1; Discharged home week 2 n=1, week 3 n=3;
[e] Data not reported for parenteral intake as 7 infants in P-PN and 6 infants in control had parenteral intake in week 2
[f] No infants had parenteral intake in week 3

Supplementary Table 3. Clinical outcomes

Outcome	P-PN (n=42) N (%)	Control (n=50) N (%)	P-value[a]
Oxygen at discharge home	0 (0.0)	2 (4.0)	0.5
Intraventricular haemorrhage	1 (2.4)	0 (0.0)	0.5
Necrotising enterocolitis	0 (0.0)	1 (2.0)	1.0

[a] Due to low counts, groups compared using Fisher's exact test

Supplementary Table 4. Peripheral intravenous cannulae

Outcome	P-PN (n=42)	Control (n=50)	Adjusted effect (95% CI) [a]	P-value [a]
Length of time peripheral IV required (days), mean, SD	5.9 (1.9)	5.7 (1.3)	1.0 (0.9, 1.2)[b]	0.5
Peripheral IV infiltration[c], n,%	24 (57)	32 (64)	0.8 (0.3, 1.8)[d]	0.5
Peripheral cannula, number required, mean, SD	2.1 (1.1)	2.2 (0.9)	1.1 (0.9, 1.3) [b]	0.3

[a] Adjusted for sex and gestational age at birth

[b] Ratio of means (95% CI)

[c] IV classification stage ≥1 (Montgomery LA, Hanrahan K, Kottman K et al. Guideline for IV infiltrations in pediatric patients. Pediatric Nursing. 1999;25(2):167-180.)

[d] Odds ratio (95% CI)

Supplementary Table 5. Mean biochemical measures by day of life and overall

Outcome	Day	P-PN (n=42)		Control (n=50)		P[a] (interaction)	Adjusted mean difference[a] (95% CI)	P[a]
		n	Mean (SD)	*n*	Mean (SD)			
Blood Urea Nitrogen, mmol/L	2	*39*	5.3 (1.8)	*43*	4.0 (2.0)		1.2 (0.7, 1.7)	<0.0001
	4	*39*	5.4 (1.6)	*45*	2.6 (1.5)		2.7 (2.1, 3.2)	<0.0001
	7	*32*	4.7 (2.0)	*43*	3.6 (1.8)		1.1 (0.2, 2.0)	0.02
	14	*27*	5.0 (1.3)	*36*	4.7 (1.6)		0.3 (-0.4, 0.9)	0.5
	21	*20*	4.1 (1.2)	*30*	3.7 (1.0)		0.5 (-0.2, 1.3)	0.1
	Overall		5.0 (1.7)		3.7 (1.8)	<0.0001		
Albumin, g/L	2	*41*	25.1 (3.1)	*44*	25.9 (3.2)			
	4	*41*	26.8 (3.8)	*45*	26.8 (3.1)			
	7	*37*	27.8 (2.5)	*46*	27.9 (2.6)			
	14	*32*	27.6 (1.9)	*37*	27.5 (2.1)			
	21	*24*	26.7 (1.7)	*33*	26.9 (1.9)			
	Overall		26.7 (3.0)		27.0 (2.7)	0.1	0.2 (-0.5, 0.8)	0.6
Triglyceride[b], mmol/L	2	*40*	1.0 (0.4)	*46*	0.6 (0.5)[c]		1.7 (1.4, 2.0)	<0.0001
	4	*41*	1.1 (0.3)	*46*	0.8 (0.3)[c]		1.4 (1.2, 1.6)	<0.0001
	7	*36*	1.0 (0.3)	*46*	1.2 (0.4)[c]		0.9 (0.7, 1.0)	0.02
	14	*32*	0.7 (0.2)	*38*	0.8 (0.3)[c]		0.9 (0.8, 1.0)	0.05
	21	*24*	0.7 (0.3)	*33*	0.8 (0.3)[c]		0.9 (0.8, 1.1)	0.4
	Overall		0.9 (0.3)		0.9 (0.4)[c]	<0.0001		
pH	2	*42*	7.4 (0.1)	*49*	7.4 (0.0)			
	4	*41*	7.4 (0.0)	*48*	7.4 (0.0)			
	7	*37*	7.4 (0.0)	*46*	7.4 (0.0)			
	14	*32*	7.4 (0.0)	*38*	7.4 (0.0)			
	21	*27*	7.4 (0.0)	*33*	7.4 (0.0)			
	Overall		7.4 (0.0)		7.4 (0.0)	0.07	-0.0 (-0.0, 0.0)	0.7
Base Excess	2	*42*	-2.1 (2.1)	*49*	-2.2 (1.7)			

Outcome	Day	P-PN (n=42)		Control (n=50)		P[a] (interaction)	Adjusted mean difference[a] (95% CI)	P[a]
		n	Mean (SD)	*n*	Mean (SD)			
	4	*41*	-2.6 (2.2)	*48*	-2.7 (1.8)			
	7	*37*	-0.7 (2.0)	*46*	-1.1 (2.6)			
	14	*32*	1.6 (1.8)	*38*	0.9 (2.0)			
	21	*27*	1.9 (1.7)	*33*	1.1 (1.8)			
	Overall		-0.7 (2.7)		-1.0 (2.5)	0.4	0.2 (-0.2, 0.7)	0.3
Blood glucose level, mmol/L	2	*42*	4.4 (1.2)	*49*	4.6 (1.1)			
	4	*42*	5.1 (1.0)	*48*	5.5 (1.4)			
	7	*38*	5.4 (1.1)	*47*	5.3 (1.2)			
	14	*32*	5.3 (1.0)	*38*	5.3 (0.8)			
	21	*27*	5.0 (0.8)	*33*	5.0 (1.1)			
	Overall		5.0 (1.1)		5.1 (1.2)	0.5	-0.0 (-0.3, 0.2)	0.8

[a] adjusted for sex, gestational age at birth and corresponding baseline measure
[b] log-transformed due to heterogeneous residual variance
[c] effects are ratio of geometric means

Supplementary Table 6. Biochemical measures outside normal clinical parameters[a]

Outcome	Day	P-PN (n=42) N (%)	Control (n=50) N (%)	P-value[b]
High blood urea nitrogen (>14.3 mmol/L)[c]	2	0 (0.0)	0 (0.0)	
	4	0 (0.0)	0 (0.0)	
	7	0 (0.0)	0 (0.0)	
	14	0 (0.0)	0 (0.0)	
	21	0 (0.0)	0 (0.0)	
	Overall	0 (0.0)	0 (0.0)	-[e]
Low serum albumin (<18 g/L)[c]	2	2 (4.8)	0 (0.0)	
	4	1 (2.4)	0 (0.0)	
	7	0 (0.0)	0 (0.0)	
	14	0 (0.0)	0 (0.0)	
	21	0 (0.0)	0 (0.0)	
	Overall	2 (4.8)	0 (0.0)	0.2
Hypertriglyceridemia (>2.25 mmol/L)[d]	2	1 (2.4)	1 (2.0)[f]	
	4	0 (0.0)	0 (0.0)	
	7	1 (2.4)	1 (2.0)[f]	
	14	0 (0.0)	0 (0.0)	
	21	0 (0.0)	0 (0.0)	
	Overall	2 (4.8)	2 (4.0)	1.0
Hyperglycemia (>8.3 mmol/L)[c]	2	1 (2.4)	0 (0.0)	
	4	0 (0.0)	1 (2.0)	
	7	1 (2.4)	2 (4.0)	
	14	1 (2.4)	0 (0.0)	
	21	0 (0.0)	0 (0.0)	
	Overall	2 (4.8)	3 (6.0)	1.0

**Outcome**	**Day**	**P-PN (n=42)** N (%)	**Control (n=50)** N (%)	**P-value**[b]
Metabolic acidosis (pH <7.25 and/or Base Excess <-5)[c]	2	3 (7.1)	2 (4.0)	
	4	4 (9.5)	2 (4.0)	
	7	0 (0.0)	3 (6.0)	
	14	0 (0.0)	0 (0.0)	
	21	0 (0.0)	0 (0.0)	
	Overall	7 (16.7)	6 (12.0)	0.6

[a] Only descriptive summary shown due to low counts

[b] from Fisher's exact test

[c] cut-off limits as used in: Osborn DA, Schindler T, Jones LJ, Sinn JKH, Bolisetty S. Higher versus lower amino acid intake in parenteral nutrition for newborn infants. Cochrane Database of Systematic Reviews. 2018; Issue 3. Art. No.: CD005949. DOI: 10.1002/14651858.CD005949.pub2.

[d] limit as used in: Kapoor V, Malviya MN, Soll R. Lipid emulsions for parenterally fed preterm infants. Cochrane Database of Systematic Reviews. 2019; Issue 6. Art. No.: CD013163. DOI: 10.1002/14651858.CD013163.pub2.

[e] no P-value obtained since no participant in the study had a high blood urea nitrogen

[f] Infant did not receive intravenous SMOFlipid
